# The Role of Prognostic Clinical-Pathological, (Immuno-) Histological, and Molecular Parameters in Pseudomyxoma Peritonei Patients Undergoing Cytoreductive Surgery and Hyperthermic Intraperitoneal Chemotherapy: A Systematic Review and Meta-Analysis

**DOI:** 10.3390/cancers18050795

**Published:** 2026-02-28

**Authors:** Jan Philipp Ramspott, Anna Kolmhofer, Lukas Schabl-Cayron, Philipp Schredl, Klaus Emmanuel, Dino Bekric, Jaroslav Presl, Andreas Pascher, Daniel Neureiter, Tarkan Jäger

**Affiliations:** 1 Department of General, Visceral and Transplant Surgery, University Hospital Muenster, 48149 Muenster, Germany; andreas.pascher@ukmuenster.de; 2 Department of Surgery, Paracelsus Medical University Salzburg, 5020 Salzburg, Austria; l.schabl-cayron@salk.at (L.S.-C.); p.schredl@salk.at (P.S.); k.emmanuel@salk.at (K.E.); j.presl@salk.at (J.P.); ta.jaeger@salk.at (T.J.); 3 Laboratory of Immunological and Molecular Cancer Research, Department of Internal Medicine III, Salzburg Cancer Research Institute, 5020 Salzburg, Austria; a.kolmhofer@crcs.at; 4 Institute of Pathology, Paracelsus Medical University Salzburg, 5020 Salzburg, Austria; dino.bekric@pmu.ac.at (D.B.); d.neureiter@salk.at (D.N.); 5 Cancer Cluster Salzburg, 5020 Salzburg, Austria; 6 Department of Biosciences, Paris Lodron University Salzburg, 5020 Salzburg, Austria

**Keywords:** pseudomyxoma peritonei, cytoreductive surgery, hyperthermic intraperitoneal chemotherapy, prognosis, risk factor, biomarker, global cancer care, oncological outcomes, cancer detection

## Abstract

**Background/Objectives**: While cytoreductive surgery (CRS) and hyperthermic intraperitoneal chemotherapy (HIPEC) significantly improve the outcome of pseudomyxoma peritonei (PMP) patients, recurrence remains a concern, highlighting the need for careful patient selection. This systematic review and meta-analysis evaluated the clinical-pathological, (immuno-) histological, and molecular prognostic factors in PMP patients undergoing CRS and HIPEC. **Methods**: Databases including PubMed, MEDLINE, Cochrane library, Embase, Scopus, and Web of Science were searched up to July 2024. Studies assessing clinical-pathological, (immuno-) histological, and molecular parameters influencing overall, progression-free, and/or disease-free survival were included. Statistical analysis was performed using R (Version 4.4.3) with random-effect models employed to address heterogeneity. Subsequent subgroup analyses were conducted to investigate potential sources of publication bias (Egger test). **Results**: A total of 16 studies involving 4009 PMP patients were included. Age, sex (male), completeness of cytoreduction score, peritoneal cancer index, tumor markers (CEA, CA 19-9, CA-125), tumor grade (high), histological subtype, and Ki-67 were significantly associated with prognosis prediction. **Conclusions**: Despite the presence of heterogeneity and publication bias, clinical-pathological and (immuno-) histological parameters were significantly associated with prognosis in PMP. The findings highlight the importance of integrating clinical-pathological and (immuno-) histological parameters in the individualized multidisciplinary management of PMP patients undergoing CRS and HIPEC. Future research should focus on incorporating molecular and genetic biomarkers to further refine patient selection and improve treatment outcomes.

## Simple Summary

Pseudomyxoma peritonei is a rare condition in which mucinous tumor cells spread throughout the abdomen, most often originating from a ruptured mucinous tumor of the appendix. The principal therapeutic approach, combining cytoreductive surgery to remove visible disease with hyperthermic intraperitoneal chemotherapy, has greatly improved patient survival. However, recurrence remains a major concern, making careful selection of patients essential. This meta-analysis synthesizes the role of prognostic clinical-pathological, (immuno-) histological, and molecular parameters in pseudomyxoma peritonei patients undergoing cytoreductive surgery and hyperthermic intraperitoneal chemotherapy. The findings emphasize the importance of using detailed tumor and patient characteristics to guide personalized, multidisciplinary treatment decisions. Looking ahead, future research should integrate molecular and genetic biomarkers to further improve patient selection and optimize treatment results for pseudomyxoma peritonei patients.

## 1. Introduction

Pseudomyxoma peritonei (PMP) is characterized by the accumulation and extensive colonization of mucinous tumor cells, frequently resulting from a perforated mucinous appendiceal tumor [1,2].

PMP is a rare disease with a recently estimated incidence of 3.2 per million and a prevalence of 22 per million in Europe [3]. Females are affected two to three times more frequently than males [4,5,6]. Although the intra-abdominal mucinous tumor masses may vary among different patients, without a therapeutic surgical approach, progressive intestinal obstruction, followed by nutritional deficiencies, will inevitably lead to the patient’s death.

Cytoreductive surgery (CRS) combined with hyperthermic intraperitoneal chemotherapy (HIPEC) is regarded as the principal therapeutic approach, significantly improving long-term prognosis in case of complete cytoreduction (completeness of cytoreduction score CC0 or CC1) [7,8,9,10,11]. CRS-HIPEC significantly improves the overall survival (OS) of PMP patients up to 196 months, with 5- and 10-year survival rates reaching up to 74% and 63%, respectively [12]. However, approximately one-quarter to nearly half of patients experience tumor recurrence and progression following CRS-HIPEC [13,14,15,16,17]. In such instances, the efficacy of repeat CRS-HIPEC is often limited, and not all patients derive benefit from the procedure, thereby necessitating meticulous patient selection. Furthermore, CRS-HIPEC is a multifaceted surgical intervention characterized by extensive resection and intricate reconstructive procedures, resulting in prolonged operative durations and potential adverse events, including intra-abdominal infections, renal and hematologic toxicity, and thromboembolic events [18,19,20]. The major complication rate is 20–34%, while perioperative mortality is reported between 1.5% and 4% in patients with PMP undergoing CRS-HIPEC. However, outcomes may vary based on patient selection and center experience [7,12]. Notably, significant postoperative complications are associated with diminished OS and disease-free survival (DFS) rates, as well as extended hospital stays [21,22].

To date, individual small-scale retrospective analyses have identified certain risk factors associated with OS, progression-free (PFS), and DFS in PMP patients undergoing CRS and HIPEC.

In the dynamic field of personalized multimodal oncological treatment, the identification of predictive clinical-pathological, (immuno-) histological, and molecular markers is crucial for effective patient selection, particularly for those who may benefit from CRS-HIPEC. This systematic review and meta-analysis aimed to synthesize and evaluate the current evidence of prognostic clinical-pathological, (immuno-) histological, and molecular parameters in pseudomyxoma peritonei patients undergoing cytoreductive surgery and hyperthermic intraperitoneal chemotherapy, thus guiding clinical decision-making.

## 2. Materials and Methods

A systematic literature review was performed in line with the Preferred Reporting Items for Systematic Reviews and Meta-Analyses (PRISMA) guidelines [23]. The study protocol has been registered in the PROSPERO international prospective register of systematic reviews (PROSPERO 2025 CRD420251148221).

### 2.1. Search Strategy

The literature search was conducted by two independent reviewers. The electronic databases PubMed, MEDLINE, Cochrane library, Embase, Scopus, and Web of Science were included for literature search. The search strategy included the following key words in adaptation to the specific syntax of each database: (HIPEC AND prognosis AND biomarker) OR (HIPEC AND prognosis AND histochemistry) OR (HIPEC AND prognosis AND immunohistochemistry) OR (HIPEC AND prognosis AND molecular pathology).

The search was performed from 1 October 2023 to 30 July 2024. Additionally, a snowball search of the included references was conducted [24]. Initially, titles and abstracts were screened by two independent reviewers according to the predefined inclusion criteria. Subsequently, a full-text evaluation was conducted, and data were extracted. In total, 16 studies were identified and included in the analysis (Table 1).

### 2.2. Inclusion and Exclusion Criteria

Following an initial literature screening of studies concerning patients with peritoneal malignancies undergoing CRS and HIPEC, only those research papers that met specific inclusion criteria were incorporated into this systematic review. The inclusion criteria were as follows: retro- and prospective studies involving patients with histologically confirmed PMP; studies evaluating the prognostic value of clinical-pathological, (immuno-) histological, and molecular markers on CRS and HIPEC efficacy based on OS, PFS, and DFS; and studies reporting survival outcomes with hazard ratios (HRs), 95% confidence intervals (CIs). The exclusion criteria encompassed studies involving tumors other than PMP, studies not explicitly assessing the impact of prognostic factors on CRS and HIPEC efficacy, studies lacking survival outcomes and adequate statistical data (e.g., HRs), and studies that did not involve surgical treatment based on the combined approach of CRS and HIPEC. Non-English articles, conference papers, unpublished works, and animal studies were also excluded from this systematic review.

### 2.3. Data Extraction

Data were extracted from each article by two independent reviewers, encompassing the following variables: The first author’s name, publication year, country, study design, sample size, patients’ baseline characteristics, and clinical-pathological, (immuno-) histological, and molecular factors associated with OS, PFS, and/or DFS prediction, as well as reported HRs, 95% CIs, and *p*-values. In instances of discrepancies, a third reviewer was consulted to reach a final decision and achieve consensus. If multiple HRs were reported for the same parameter, the HR corresponding to the highest-risk category (the most clinically relevant contrast) was selected for statistical analysis; this situation occurred in only one study [25].

### 2.4. Outcome

The primary objective was to assess the impact of clinical-pathological, (immuno-) histological, and molecular parameters influencing OS, PFS, and/or DFS in patients suffering from PMP undergoing CRS and HIPEC.

### 2.5. Statistical Analysis

Statistical analysis was performed using R (Version 4.4.3) with the metafor package [41]. For statistical analysis, all parameters influencing OS, PFS, and/or DFS were categorized as follows: age, CC score, sex, PCI, performance status, symptoms, therapy, tumor marker (category: clinical-pathological); grading, histology, and immunohistochemistry (category: [immuno-] histological); and molecular pathology (category: molecular).

#### Statistical Heterogeneity and Publication Bias

Survival data were analyzed utilizing HRs and their corresponding 95% CIs through multivariate regression techniques. Statistical heterogeneity among studies was assessed using I^2^ statistics, with thresholds for low, moderate, and high heterogeneity set at 25%, 50%, and 75%, respectively [42]. To accommodate potential variability across studies, a random-effects model and Restricted Maximum Likelihood (REML) were consistently utilized, irrespective of the degree of heterogeneity. This approach was adopted to more accurately capture the true variations among studies and to extend our conclusions to a broader patient population. Furthermore, to obtain a more conservative estimate of the effect size, the Hartung–Knapp–Sidik–Jonkman (HKSJ) adjustment was applied for indicators with greater heterogeneity to correct the test statistics and CIs.

Subsequent subgroup analyses were conducted to investigate potential sources of publication bias. The Egger test, as an indicator of funnel plot asymmetry, was employed for each category to assess the correlation between the effect estimate and its variance, with a *p* value of less than 0.1 indicating a significant difference [43]. Statistical significance was set at a threshold of *p* < 0.05. The trim-and-fill method was applied as a sensitivity analysis to further estimate the potential impact of publication bias and to provide bias-adjusted pooled estimates.

### 2.6. Methodological Quality and Grade of Evidence Assessment

The quality and the grade of evidence of the included studies were assessed by two independent investigators based on the Newcastle Ottawa Scale (NOS) [44] and Grading of Recommendations, Assessment, Development and Evaluation (GRADE) Working Group grades of evidence (high, moderate, low, very low quality) [45], respectively. Concerning NOS scaling, all studies were evaluated based on patient selection, comparability of cohorts based on the design or analysis, and assessment of measured outcomes. The studies were then rated as high (7–9), moderate (4–6), or low quality (0–3).

## 3. Results

### 3.1. Study Selection Process and Characteristics of Included Studies

**Figure 1:**
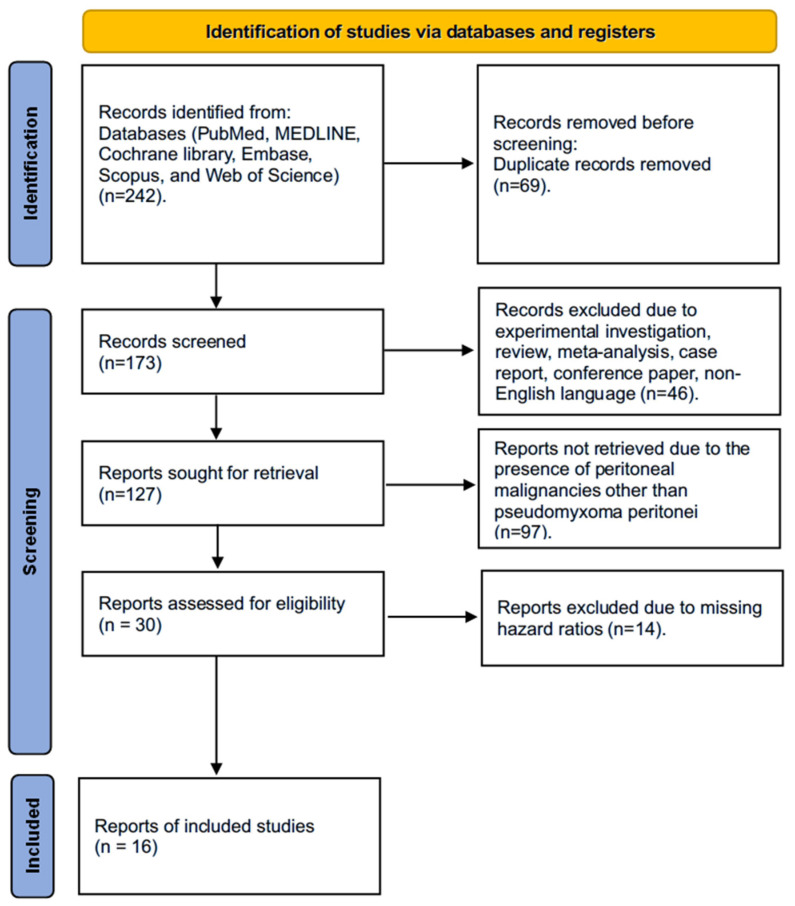
PRISMA flowchart showing the article selection process.

We identified 242 studies using the search strategy. Finally, this meta-analysis includes a total of 4009 patients diagnosed with PMP, derived from 16 studies published between 2002 and 2023, following the application of inclusion criteria. The detailed PRISMA flowchart illustrating the article selection process is presented in Figure 1. Excluded studies due to missing HRs for OS, PFS, or DFS are presented in Table S1.

Among the 16 studies included, all examined the impact of clinical-pathological parameters, ten assessed histological parameters, two evaluated immunohistochemical parameters, and one investigated molecular parameters on the prognosis of CRS-HIPEC in PMP patients. Twelve studies reported OS, while six studies provided data on PFS and DFS, respectively. The female-to-male ratio was approximately 1:1.5, with a median age of 51 years. Most of the included studies (n = 15) were retrospective cohort studies. No randomized controlled trial was identified. Most studies were conducted in Europe (n = 12). Further details of the studies included are presented in Table 1. Detailed surgical and perioperative characteristics of the included studies are described in Table 2.

### 3.2. Study Quality and Grade of Evidence Assessment

Based on the NOS quality assessment, most studies were of moderate quality. Only one study was of high quality (Table S2). The majority of the studies’ evidence was of moderate quality; however, one study demonstrated high quality (Table S3).

### 3.3. Association of Clinical-Pathological and Histological Parameters with OS in Univariate Analysis

Four studies investigated the correlation between clinical-pathological and histological parameters and OS through univariate analysis [25,26,30,31].

The pooled analysis of all included studies indicated a strong correlation between clinical-pathological parameters and OS prediction (HR, 1.95; 95% CI: 1.51–2.50; *p* < 0.001), despite high heterogeneity (I^2^ = 99.98%). Additionally, histological parameters demonstrated a strong association with OS prediction (HR, 4.50; 95% CI: 3.33–6.07; *p* < 0.001) with low heterogeneity (I^2^ = 0.00%).

**Figure 2:**
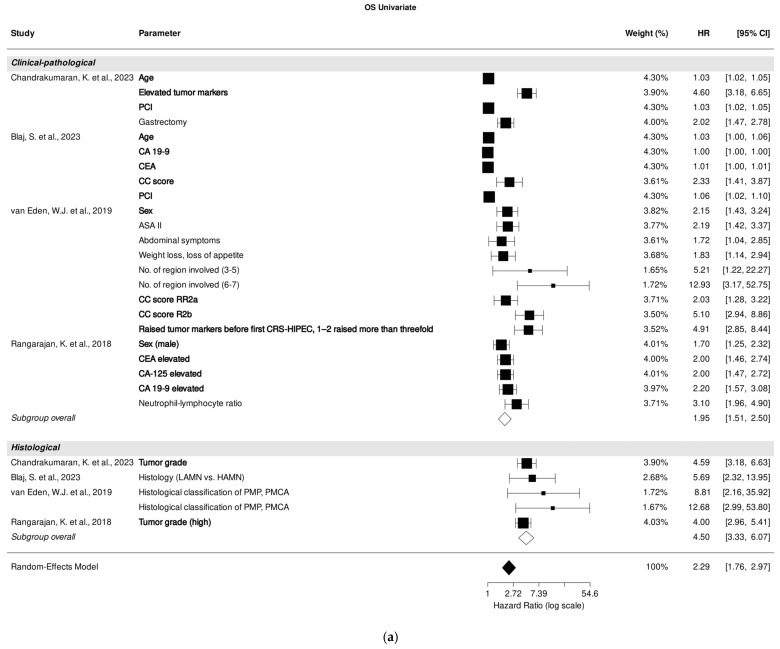

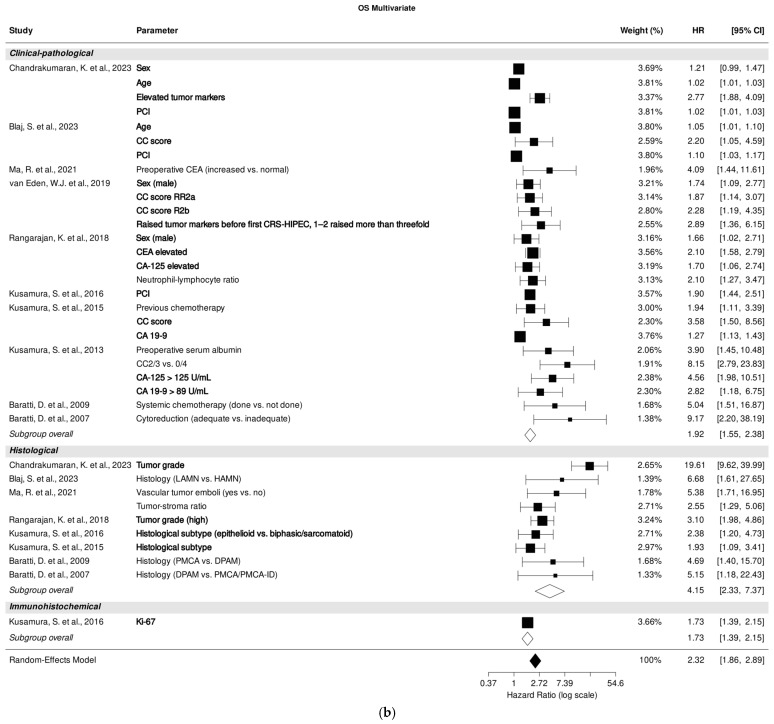
Forest plot of the association between clinical-pathological, histological, and immunohistochemical parameters with overall survival in univariate (**a**) and multivariate (**b**) analysis. Most relevant parameters are shown in bold (based on HRs, weights, and the number of mentions across the different studies). HR, hazard ratio; CI, confidence interval.

Within the studies focusing on clinical-pathological parameters, age [25,26], male sex [30,31], CC score [26,30], PCI [25,26], and tumor markers carcinoembryonic antigen (CEA), cancer antigen (CA)-125, and CA 19-9 [25,26,30,31] emerged as the most significant predictors of OS in univariate analysis. Regarding studies that investigated histological parameters, high tumor grade was identified as having the most substantial effect on OS prediction [25,31] (Figure 2a).

### 3.4. Association of Clinical-Pathological, Histological, and Immunohistochemical Parameters with OS in Multivariate Analysis

Ten studies included in this analysis examined the correlation between clinical-pathological parameters and OS through multivariate analysis [25,26,28,30,31,33,34,35,37,39]. Eight studies focused on the correlation between histological parameters and OS [25,26,28,31,33,34,37,39]. Additionally, one study examined immunohistochemical parameters [33].

A pooled analysis of all included studies revealed a strong correlation between clinical-pathological parameters and OS prediction (HR, 1.92; 95% CI: 1.55–2.38; *p* < 0.001), despite high heterogeneity (I^2^ = 99.69%). Histological parameters were also strongly associated with OS prediction (HR, 4.15; 95% CI: 2.33–7.37; *p* < 0.001), with moderate heterogeneity (I^2^ = 74.09%). Immunohistochemical parameters showed a correlation with OS prediction as well (HR, 1.73; 95% CI: 1.39–2.15; *p* < 0.001).

Among the studies examining the role of clinical-pathological parameters, factors such as age [25,26], male sex [25,30,31], CC score [26,30,34], PCI [25,26,33], and tumor markers CEA, CA-125, and CA 19-9 [25,30,31,34,35] were most significantly associated with OS prediction in multivariate analysis.

Furthermore, high tumor grade [25,31], histological subtype [33,34], and Ki-67 [33] revealed the highest impact in multivariate OS prediction regarding (immuno-) histological parameters (Figure 2b).

### 3.5. Association of Clinical-Pathological, Histological, and Molecular Parameters with PFS in Univariate Analysis

Three studies investigated the correlation between clinical-pathological parameters [26,27,32], one study [26] focused on the correlation between histological parameters, while another study examined the correlation between molecular parameters [32] with PFS in univariate analysis.

**Figure 3:**
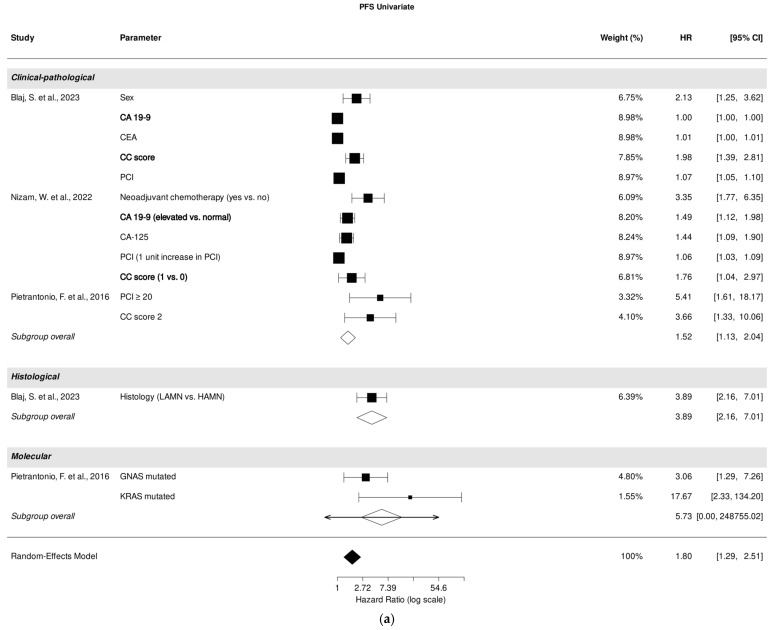

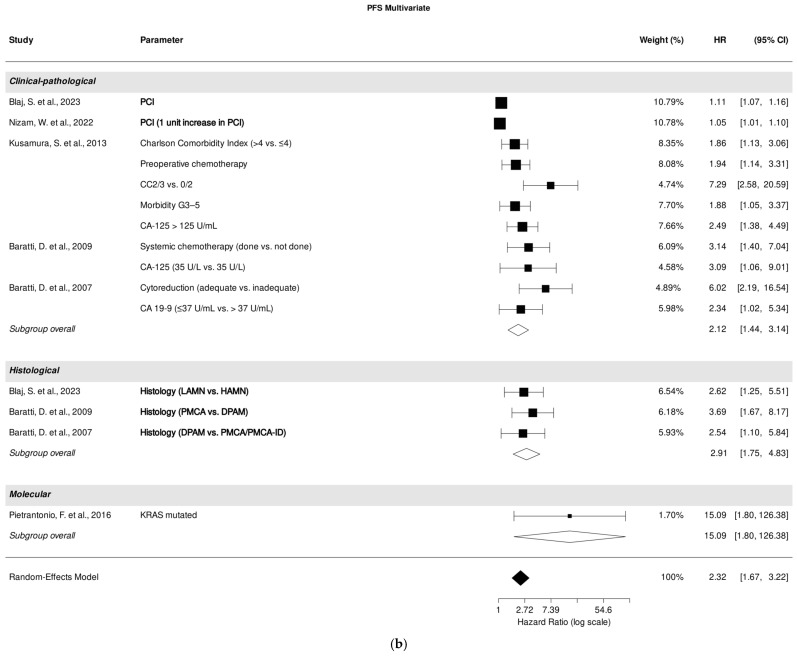
Forest plot of the association between clinical-pathological, histological, and molecular parameters with progression-free survival in univariate (**a**) and multivariate (**b**) analysis. Most relevant parameters (based on HRs, weights, and the number of mentions across the different studies) are shown in bold. HR, hazard ratio; CI, confidence interval.

A pooled analysis of all included studies revealed that clinical-pathological parameters were associated with PFS prediction (HR, 1.52; 95% CI: 1.13–2.04; *p* = 0.01), despite high heterogeneity (I^2^ = 99.99%). Histological parameters demonstrated a strong association with PFS prediction (HR, 3.89; 95% CI: 2.16–7.01; *p* < 0.001). Among all included studies examining the role of clinical-pathological parameters, CC score [26,27], PCI [26,27], and tumor marker CA 19-9 [26,27] were most significantly associated with PFS prediction (Figure 3a).

### 3.6. Association of Clinical-Pathological, Histological, and Molecular Parameters with PFS in Multivariate Analysis

Five studies investigated the correlation between clinical-pathological parameters [26,27,35,37,39], while three studies [26,37,39] focused on the correlation between histological parameters. Additionally, one study [32] examined the correlation between molecular parameters and PFS through multivariate analysis. A pooled analysis of all included studies demonstrated a strong association between clinical-pathological parameters and PFS prediction (HR, 2.12; 95% CI: 1.44–3.14; *p* = 0.002), despite high heterogeneity (I^2^ = 98,29%). Histological parameters also revealed a strong association with PFS prediction (HR, 2.91; 95% CI: 1.75–4.83; *p* = 0.012) with a low heterogeneity (I^2^ = 0.00%).

Within the analyzed studies, PCI emerged as the most significant clinical-pathological parameter associated with PFS prediction in multivariate analysis [26,27]. Additionally, histological subtypes [26,37,39] were also significantly correlated with PFS prediction (Figure 3b).

### 3.7. Association of Clinical-Pathological and Histological Parameters with DFS in Univariate Analysis

Seven studies [25,29,30,31,36,38,40] investigated the association between clinical-pathological parameters and DFS through univariate analysis, while three studies focused on histological parameters [25,30,31].

**Figure 4:**
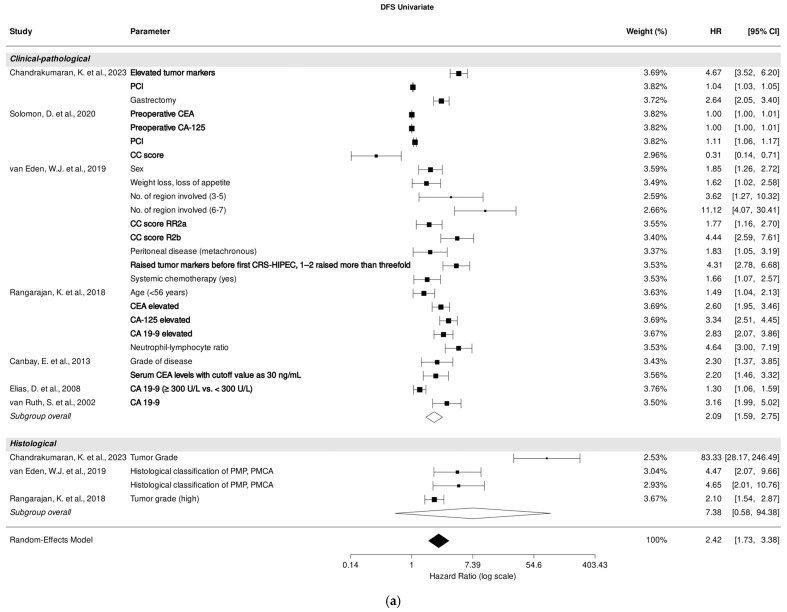

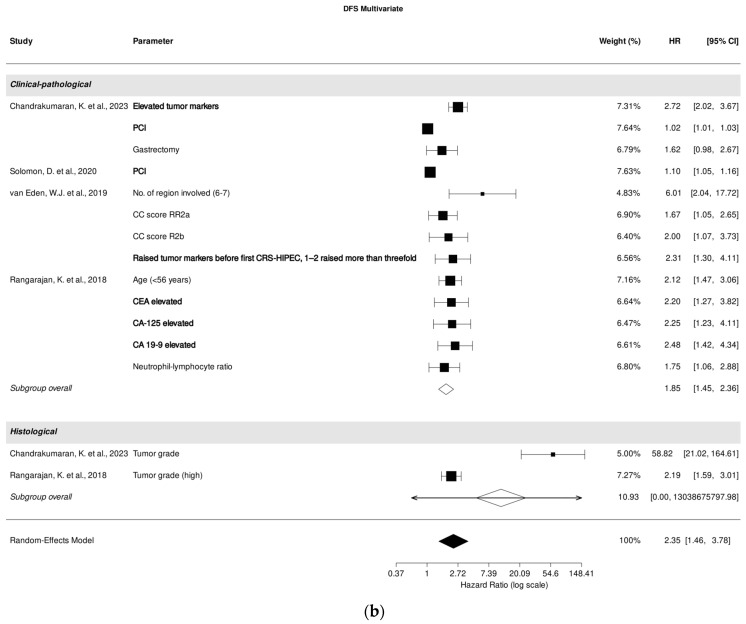
Forest plot of the association between clinical-pathological and histological parameters with disease-free survival in univariate (**a**) and multivariate (**b**) analysis. Most relevant parameters (based on HRs, weights, and the number of mentions across the different studies) are shown in bold. HR, hazard ratio; CI, confidence interval.

A pooled analysis of all included studies demonstrated a strong association between clinical-pathological (HR, 2.09; 95% CI: 1.59–2.75; *p* < 0.001) and DFS prediction, despite high heterogeneity (I^2^ = 99.99%). PCI [25,29], CC score [29,30], and tumor markers CEA, CA-125, and CA 19-9 [25,29,30,31,36,38,40] were most significantly associated with DFS prediction in univariate analysis (Figure 4a).

### 3.8. Association of Clinical-Pathological and Histological Parameters with DFS in Multivariate Analysis

Four studies [25,29,30,31] investigated the correlation between clinical-pathological parameters, while two studies [25,31] focused on the correlation between histological parameters and DFS in multivariate analysis. A pooled analysis of all included studies revealed an association between clinical-pathological parameters and DFS prediction (HR, 1.85; 95% CI: 1.45–2.36; *p* < 0.001), despite high heterogeneity (I^2^ = 96.89%). Among the studies assessing the role of clinical-pathological parameters, PCI [25,29] and tumor markers CEA, CA-125, and CA 19-9 [25,30,31] were most significantly associated with DFS prediction in multivariate analysis (Figure 4b).

The key findings of this meta-analysis are summarized in Table 3.

### 3.9. Publication Bias

#### 3.9.1. Publication Bias OS Univariate Analysis

In studies investigating the association between clinical-pathological and histological parameters and OS through univariate analysis, the asymmetrical distribution of studies suggested a significant risk of publication bias. This was corroborated by Egger’s regression test with a *p* value of <0.0001 (Supplementary Figure S1a). The trim-and-fill method identified eight missing studies on the left side of the funnel plot, suggesting a potential overestimation of effect sizes due to small study effects (Supplementary Figure S1b; Table S4).

#### 3.9.2. Publication Bias OS Multivariate Analysis

In studies examining the association between clinical-pathological, histological and immunohistochemical parameters and OS through multivariate analysis, the asymmetrical distribution of studies indicated the presence of publication bias, which was supported by Egger’s regression test (*p* < 0.0001) (Supplementary Figure S1c). The trim-and-fill method identified 14 missing studies on the left side of the funnel plot, suggesting a potential overestimation of effect sizes due to small study effects (Supplementary Figure S1d; Table S4).

#### 3.9.3. Publication Bias PFS Univariate Analysis

In studies investigating the association between clinical-pathological, histological, and molecular parameters and PFS through univariate analysis, the asymmetrical distribution of studies indicated the presence of publication bias, which was further corroborated by Egger’s regression test with a *p* value of <0.0001 (Supplementary Figure S2a). The trim-and-fill method identified six missing studies on the left side of the funnel plot, suggesting a potential overestimation of effect sizes due to small study effects (Supplementary Figure S2b, Table S4).

#### 3.9.4. Publication Bias PFS Multivariate Analysis

In studies examining the association between clinical-pathological, histological, and molecular parameters and PFS through multivariate analysis, the asymmetrical distribution of studies indicated the presence of publication bias, which was further corroborated by Egger’s regression test with a *p* value of <0.0001 (Supplementary Figure S2c). The trim-and-fill method identified six missing studies on the left side of the funnel plot, suggesting a potential overestimation of effect sizes due to small study effects (Supplementary Figure S2d; Table S4).

#### 3.9.5. Publication Bias DFS Univariate Analysis

In studies investigating the association between clinical-pathological and histological parameters and DFS through univariate analysis, the asymmetrical distribution of studies indicated the presence of publication bias, which was further corroborated by Egger’s regression test with a *p* value of <0.0001 (Supplementary Figure S3a). Trim-and-fill analyses showed no imputed studies, implying a lower likelihood of publication bias, but still a high heterogeneity (Table S4).

#### 3.9.6. Publication Bias DFS Multivariate Analysis

In studies examining the association between clinical-pathological and histological parameters and DFS through multivariate analysis, the asymmetrical distribution of studies indicated the presence of publication bias, which was further corroborated by Egger’s regression test with a *p* value of <0.0001 (Supplementary Figure S3b). Trim-and-fill analyses showed no imputed studies, implying a lower likelihood of publication bias, but still a high heterogeneity (Table S4).

## 4. Discussion

PMP is a rare malignancy frequently resulting from a perforated mucinous appendiceal tumor [1,2]. CRS and HIPEC remain the main therapeutic interventions. Nevertheless, there is potential to further refine eligibility criteria, as post-surgical tumor recurrence and progression significantly impact long-term survival [7,8,9,10,11]. Until now, the traditional criteria such as operability (based on patient’s ability to tolerate major surgery) and resectability (based on the extent and distribution of peritoneal disease) seem to appear insufficient.

In this study, we provide one of the first comprehensive analyses of predictive markers affecting OS, PFS, and DFS following CRS and HIPEC in patients with PMP. The pooled analysis, including 16 studies, identified the following risk factors: three patient-related risk factors (age, male sex, and PCI), three tumor-markers (CEA, CA-125, CA 19-9), two histological parameters (high tumor grade and histological subtype), one immunohistochemical parameter (Ki-67), and one surgery-related risk factor (CC score).

### 4.1. Prognostic Factors on CRS and HIPEC Efficacy

#### 4.1.1. Clinical-Pathological Risk Factors

##### Patient-Related Risk Factors

In this study, age, male sex, PCI, and tumor markers CEA, CA-125, and CA 19-9 emerged as the most significant factors associated with prognosis. Epidemiological data indicate that both the prevalence and incidence of PMP increase with advancing age, with the highest rates observed in the oldest demographic groups. In a nationwide Chinese cohort, both prevalence and incidence were found to rise across age strata, reaching a peak in individuals over 80 years of age [46]. Moreover, advanced age is directly correlated with an increase in comorbidities and a diminished capacity for recovery following major surgical procedures, potentially negating any long-term survival benefits conferred by CRS and HIPEC [47]. In this context, the age threshold most consistently associated with increased postoperative complications after CRS-HIPEC in PMP patients is ≥65 years [7,25,48,49,50].

The association of male patients with OS prediction aligns with findings from one of the largest studies evaluating outcomes and long-term survival following CRS-HIPEC in PMP patients [12] and a recent meta-analysis [51]. The existing literature suggests that male patients may exhibit symptom onset at a more advanced stage compared to female patients [4]. So far, there is no definitive hypothesis to explain this discrepancy. It is possible that male patients may delay seeking medical attention and overlook symptoms, a pattern observed in other cancers, such as colorectal cancer [52,53].

Furthermore, the PCI score, as the most widely utilized tool for determining the extent of peritoneal disease, has been associated with OS, PFS, and DFS prediction. A practical prognostic dichotomy at a PCI of approximately ≤21 for low-grade and ≤25 for high-grade tumors is currently supported [54]. It is acknowledged that histologic grade and the completeness of cytoreduction influence risk, and that selected patients with a high PCI may still derive benefit from CRS and HIPEC when complete cytoreduction (CC0/1) is achievable [25,26,54,55]. This aligns with a recent meta-analysis, which indicates that a PCI exceeding 20 points is associated with poor surgical outcomes and a higher risk of recurrence [51]. In a large international registry, Chua et al. demonstrated that a high PCI is an independent predictor of poor PFS [12].

Our meta-analysis indicated that tumor markers CEA, CA-125, and CA 19-9 are associated with OS, PFS, and DFS prediction, which is in line with a recent meta-analysis [51]. In the context of PMP, these tumor markers could be crucial for baseline risk stratification assessing the predictability of resection. Regarding the initial evaluation of disease burden and resectability, elevated preoperative levels of CEA, CA 19-9, and CA-125 correlate with higher PCI and lower likelihood of achieving complete cytoreduction [27,35,39,56,57]. Notably, CA 19-9 frequently demonstrates the strongest association with earlier progression, while both CA-125 and CA 19-9 are linked to inferior OS in several cohorts [26,35,39,40,51,56,57,58,59,60,61].

##### Surgery-Related Risk Factors

Achieving complete cytoreduction (CC0/1) is the primary determinant of long-term survival, with HIPEC commonly integrated [12]. Patients with CCR2 or CCR3, characterized by gross residual disease, exhibit a 5-year survival rate of 24% in contrast to 85% in CCR0 patients [12]. In this study, complete/incomplete cytoreduction was associated with OS, PFS, and DFS prediction. These results are in line with a recent meta-analysis by Wei et al. who identified the CC score as a risk factor associated with surgery [51]. Even histologically bland or low-grade appearance PMP often necessitates highly aggressive and extensive surgical intervention due to the biological behavior of PMP, which is not reflected by its histology [62].

#### 4.1.2. (Immuno-) Histological Parameters

Concerning histological parameters, both high tumor grade and histological subtype exhibited the most significant associations with prognosis, including OS and PFS prediction. The Peritoneal Surface Oncology Group International (PSOGI) consensus categorizes PMP disease into acellular mucin (acellular mucinous peritoneal deposits), low-grade mucinous carcinoma peritonei/disseminated peritoneal adenomucinosis (DPAM), high-grade mucinous carcinoma peritonei/peritoneal mucinous carcinomatosis (PMCA), and high-grade mucinous carcinoma peritonei with signet ring cells/peritoneal mucinous carcinomatosis with signet ring cells (PMCA-S) [63,64], in alignment with the World Health Organization (WHO) two-tier system (low vs. high grade) [65]. Recent studies involving CRS and HIPEC indicate that survival outcomes are most favorable for acellular mucin, moderate for low-grade in comparison with less favorable for high-grade, and finally to poorest in the presence of signet ring cells [12,63,64]. Multivariable analyses consistently identified the histologic grade of the peritoneum as an independent predictor of OS, with risk increasing from low-grade to high-grade to signet ring cell categories [25,26,66].

In the context of immunohistochemical parameters, our meta-analysis identified Ki-67 as the sole predictor of OS in patients with PMP undergoing CRS and HIPEC. For high-grade PMP, a Ki-67 proliferation index threshold of approximately 15% has been shown to effectively stratify prognosis. Patients with high-grade PMP exhibiting a Ki-67 index exceeding 15% experience significantly poorer OS and DFS compared to those with an index of 15% or less. This finding has prompted a proposed modification of the PSOGI classification, which further subdivides high-grade PMP based on Ki-67 levels [67,68].

#### 4.1.3. Molecular Parameters

Our meta-analysis did not identify any significant prognostic molecular markers; however, this likely reflects the limited availability of data rather than a true lack of biological relevance. Molecular markers in PMP primarily function to elucidate pathogenesis and provide prognostic stratification. The genes most mutated in PMP are KRAS and GNAS, with KRAS mutations occurring in 38–100% of cases and GNAS in 17–100%, often appearing together. These mutations are crucial to the pathogenesis of PMP and are linked to mucin overproduction through the PKA pathway, a characteristic feature of the disease [32,69,70,71,72,73,74]. So far, no clinical study has reported data on KRAS inhibition, but in vitro and in vivo models have shown promising results in targeting KRAS in PMP [75,76].

### 4.2. Strengths and Limitations of the Study

In contrast to two earlier systematic reviews [77,78] and one recent meta-analysis [51], our meta-analysis neither assessed the efficacy or safety of cytoreductive surgery and HIPEC in patients with pseudomyxoma peritonei nor focused exclusively on clinical prognostic factors. Instead, it comprehensively integrated (immuno-) histological and molecular parameters, thereby enabling a broader and more in-depth evaluation of prognostic determinants.

However, it is important to acknowledge certain limitations inherent in our study. Firstly, most of the studies included were retrospective in nature, which may introduce selection bias and impact the overall quality of the evidence. To date, no randomized controlled trials have been conducted. Due to high heterogeneity, the pooled estimates should be interpreted with caution.

Secondly, the results of the meta-analysis may be influenced by the variability in patient selection criteria across the included studies, such as age, systemic anticancer treatments, comorbidities, and performance status.

Thirdly, this review revealed that many studies report only a limited set of key surgical characteristics, and there is substantial variability in the HIPEC procedures themselves, particularly regarding duration, temperature, drug choice, and drug concentration. The studies included were conducted over a span of more than two decades, which may contribute to a significant publication bias. During this period, there have been changes in surgical teams, HIPEC regimens, and the classification and awareness of PMP. Lastly, the restriction to English-language studies, predominantly conducted in Europe, and the use of different statistical methods in the included studies may further limit the reliability and generalizability of our meta-analysis.

## 5. Conclusions

Despite significant heterogeneity and possible selection bias due to mostly retrospective included studies, our meta-analysis primarily identified clinical-pathological parameters such as age, male sex, PCI, tumor markers, high tumor grade, histological subtype, and CC score as the most pertinent indicators associated with OS, PFS, and DFS prediction in PMP patients undergoing CRS and HIPEC. Currently, immunohistochemical and molecular markers only play a minor prognostic role. Effective individualized multidisciplinary management of PMP requires the integration of molecular and genetic biomarkers [79]; therefore, the current scarcity of molecular studies must be urgently addressed through well-designed, adequately powered investigations of markers such as KRAS and GNAS to clarify their prognostic and potential therapeutic relevance.

## Figures and Tables

**Table 1:** Basic clinical characteristics of the included studies of the meta-analysis.

No.	Author	Year	Country	Study Design	Clinical Investigation	Number of Patients [n] (Female [n]/ Male [n])	Age [Years] Mean/ Median	OS/PFS/ DFS	Biomarker CP/H/IHC/MOL
1	Chandrakumaran, K. et al. [25]	2023	United Kingdom	Cohort study	R	1102 (710/392)	n.a./57	1/0/1	1/1/0/0
2	Blaj, S. et al. [26]	2023	Germany	Cohort study	R	193 (93/100)	n.a./61	1/1/0	1/1/0/0
3	Nizam, W. et al. [27]	2022	USA	Cohort study	R	264 (162/102)	n.a/57	1/1/0	1/0/0/0
4	Ma, R. et al. [28]	2021	China	Cohort study	R	50 (23/27)	n.a./55	1/0/0	1/1/0/0
5	Solomon, D. et al. [29]	2020	USA	Cohort study	R	156 (90/66)	n.a/53	0/0/1	1/0/0/0
6	Van Eden, W.J. et al. [30]	2019	Netherlands	Cohort study	R	225 (159/66)	n.a./54.5	1/0/1	1/1/0/0
7	Rangarajan, K. et al. [31]	2018	United Kingdom	Cohort study	R	699 (444/255)	n.a./56	1/0/1	1/1/0/0
8	Pietrantonio, F. et al. [32]	2016	Italy	Cohort study	R	40 (21/19)	n.a./52	0/1/0	1/0/0/1
9	Kusamura, S. et al. [33]	2016	Italy	Cohort study	R	117 (50/67)	n.a./60.5	1/0/0	1/1/1/0
10	Kusamura, S. et al. [34]	2015	Italy	Cohort study	R	226 (127/99)	54/n.a.	1/0/0	1/1/0/0
11	Kusamura, S. et al. [35]	2013	Italy	Cohort study	R	156 (96/60)	53.5/n.a.	1/1/0	1/0/0/0
12	Canbay, E. et al. [36]	2013	Japan	Cohort study	R	449 (245/204)	n.a./55.4	0/0/1	1/0/0/0
13	Baratti, D. et al. [37]	2009	Italy	Cohort study	R	102 (58/44)	n.a./53.5	1/1/0	1/1/1/0
14	Elias, D. et al. [38]	2008	France	Cohort study	R	105 (68/37)	48.1/n.a.	1/0/1	1/1/0/0
15	Baratti, D. et al. [39]	2007	Italy	Cohort study	P	62 (27/35)	n.a./55.5	1/1/0	1/1/0/0
16	Van Ruth, S. et al. [40]	2002	Netherlands	Cohort study	R	63 (n.a./n.a.)	n.a./n.a.	0/0/1	1/0/0/0
Summary					15/1 ^1^	4009 ^1^ (2373/1573) ^1^ 250.6 ^2^ (158.2/104.9) ^2^	51.0/55.4 ^2^	12/6/6 ^1^	16/10/2/1 ^1^

CP, clinical-pathological; H, histological; IHC, immunohistochemical; MOL, molecular; n.a., not applicable; R, retrospective; P, prospective; OS, overall survival; PFS, progression-free survival; DFS, disease-free survival; ^1^: Sum; ^2^: Mean.

**Table 2:** Surgical and perioperative characteristics of the included studies of the meta-analysis.

No.	Study	PCI	CC Score	HIPEC Procedure	HIPEC Duration [min]	HIPEC Temperature [°C]	HIPEC Drug (Concentration)	HIPEC Perfusate Volume [l]	Operative Time [min]	Previous Systemic Chemo- therapy
1	Chandrakumaran, K. et al. 2023 [25]	21 ^1^	-	-	60	42–43	Mitomycin C (10 mg/m^2^)	-	-	-
2	Blaj, S. et al. 2023 [26]	-	0: 163 1: 22 2: 8	Closed	30–90	-	Mitomycin C, Oxaliplatin	-	-	-
3	Nizam, W. et al. 2022 [27]	14 ^1^	0: 143 1: 78 2: 11 3: 8	-	-	-	Mitomycin C, Cisplatin, Carboplatin, Oxaliplatin, Melphalan	-	-	Yes: 25 No: 239
4	Ma, R. et al. 2021 [28]	36 ^1^	0–1: 18 2–3: 32	Open	60	42.5–43.5	Cisplatin (120 mg) and Docetaxel (120 mg)	3	-	Yes: 23 No: 27
5	Solomon, D. et al. 2020 [29]	18 ^1^	0: 123 1: 33	Closed	90	41–43	Mitomycin C (40 mg), Carboplatin	-	-	Yes: 11
6	Van Eden, W.J. et al. 2019 [30]	-	R1, R2a, R2b	-	90	40–43	Mitomycin C (35 mg/m^2^), Oxaliplatin (460 mg/m^2^)	-	-	Yes: 4 No: 221
7	Rangarajan, K. et al. 2018 [31]	-	-	Open	60	42–43	Mitomycin C (10 mg/m^2^), 5-FU (15 mg/kg) ^3^	-	-	-
8	Pietrantonio, F. et al. 2016 [32]	27 ^1^	0: 18 1: 17 2: 5	Closed	-	-	Mitomycin C, Cisplatin	-	-	Yes: 7 No: 33
9	Kusamura, S. et al. 2016 [33]	21 ^1^	0–1: 94 2–3: 23	Closed	-	42–43	Cisplatin (42 mg/L of perfusate) and Doxorubicin (15 mg/L), Cisplatin (42 mg/L of perfusate) and Mitomycin C (3.3 mg/m^2^/L of perfusate)	-	600 ^2^	Yes: 49 No: 68
10	Kusamura, S. et al. 2015 [34]	22 ^2^	0–1: 210 2–3: 116	Closed	-	42–43	Cisplatin (25 mg/m^2^/L), Mitomycin C (3.3 mg/m^2^/L)	-	608 ^2^	Yes: 54 No: 171
11	Kusamura, S. et al. 2013 [35]	22.6 ^2^	Optimal: 142, incomplete: 14	Closed	-	42–43	Cisplatin (25 mg/m^2^/L), Mitomycin C (3.3 mg/m^2^/L)	-	592 ^2^	Yes: 37 No: 119
12	Canbay, E. et al. 2013 [36]	-	0: 193 1: 52 2: 35 3: 169	Open	60	43	Mitomycin C 20 mg	4	-	-
13	Baratti, D. et al. 2009 [37]	21.8 ^2^	0: 35 1: 67	Closed	60	42.5	Cisplatin (25 mL/m^2^/L of perfusate) and Mitomycin C (3.3 mg/m^2^/L of perfusate)	4–6	-	Yes: 22 No: 80
14	Elias, D. et al. 2008 [38]	-	0: 105	Open	30–60	41–43	Oxaliplatin (360 mg/m^2^) and Irinotecan (360 mg/m^2^); Mitomycin C, Oxaliplatin (460 mg/m^2^ in 2 L/m^2^)	-	529 ^2^ 517 ^1^	Yes: 90 No: 15
15	Baratti, D. et al. 2007 [39]	-	0–1: 53 2–3: 9	Closed	60	42.5	Cisplatin (25 mL/m^2^/L of perfusate) and Mitomycin C (3.3 mg/m^2^/L of perfusate)	4–6	-	-
16	Van Ruth, S. et al. 2002 [40]	-	-	-	90	40–41	Mitomycin C (35 mg/m^2^)	-	-	-

PCI: peritoneal cancer index; CC: completeness of cytoreduction; HIPEC: hyperthermic intraperitoneal chemotherapy. ^1^: Median; ^2^: Mean; ^3^: Administered as early postoperative intraperitoneal chemotherapy for 3–5 days.

**Table 3:** Key findings overview of this study: Significant clinical-pathological, (immuno-) histological, and molecular parameters associated with overall, progression-free, and disease-free survival prediction in univariate and multivariate analysis. HR, hazard ratio; CI, confidence interval; OS, overall survival; PFS, progression-free survival; DFS, disease-free survival; CC, completeness of cytoreduction; PCI, peritoneal cancer index; CA, cancer antigen.

	Clinical-Pathological Parameters	Histological Parameters	Immunohistochemical Parameters
**OS** **univariate**	HR, 1.95; 95% CI: 1.51–2.50; *p* < 0.001 Age, sex, CC score, PCI, tumor markers	HR, 4.50; 95% CI: 3.33–6.07; *p* < 0.001 Tumor grade	-
**OS** **multivariate**	HR, 1.92; 95% CI: 1.55–2.38; *p* < 0.001 Age, sex, CC score, PCI, tumor markers	HR, 4.15; 95% CI: 2.33–7.37; *p* < 0.001 Tumor grade, Histological subtype	HR, 1.73; 95% CI: 1.39–2.15; *p* < 0.001 Ki-67
**PFS** **univariate**	HR, 1.52; 95% CI: 1.13–2.04; *p* = 0.01 CC score, PCI, CA 19-9	-	-
**PFS** **multivariate**	HR, 2.12; 95% CI: 1.44–3.14; *p* = 0.002 PCI	HR, 2.91; 95% CI: 1.75–4.83; *p* = 0.012 Histological subtype	-
**DFS** **univariate**	HR, 2.09; 95% CI: 1.59–2.75; *p* < 0.001 PCI, CC score, tumor markers	-	-
**DFS** **multivariate**	HR, 1.85; 95% CI: 1.45–2.36; *p* < 0.001 PCI, tumor markers	-	-

## Data Availability

Data for the systematic literature review and meta-analysis were obtained from published sources. Data are available upon request.

## Supplementary Materials

The following supporting information can be downloaded at: https://www.mdpi.com/article/10.3390/cancers18050795/s1, Figure S1: Funnel plot of studies examining the association between clinical-pathological, histological, and immunohistochemical parameters with overall survival in univariate (a), trim-and-fill adapted univariate (b), multivariate (c), and trim-and-fill adapted multivariate (d) analysis; Figure S2: Funnel plot of studies examining the association between clinical-pathological, histological, and molecular parameters with progression-free survival in univariate (a), trim-and-fill adapted univariate (b), multivariate (c), and trim-and-fill adapted multivariate (d) analysis; Figure S3: Funnel plot of studies examining the association between clinical-pathological and histological parameters with disease-free survival in univariate (a) and multivariate (b) analysis; Table S1: Excluded studies due to missing HRs for OS, PFS, or DFS; Table S2: The quality of the included studies (Newcastle-Ottawa Quality Assessment Scale); Table S3: Grading of Recommendations, Assessment, Development and Evaluation (GRADE) Working Group grades of evidence of the included studies; Table S4: Trim-and-fill sensitivity analysis results.

## Abbreviations

The following abbreviations are used in this manuscript:

CA-125	Cancer antigen 125
CC	Completeness of cytoreduction score
CEA	Carcinoembryonic antigen
CI	Confidence interval
CRS	Cytoreductive surgery
DFS	Disease-free survival
DPAM	Disseminated peritoneal adenomucinosis
GRADE	Grading of Recommendations, Assessment, Development and Evaluation
HIPEC	Hyperthermic intraperitoneal chemotherapy
HKJS	Hartung–Knapp–Sidik–Jonkman
HR	Hazard ratio
NOS	Newcastle Ottawa Scale
OS	Overall survival
PCI	Peritoneal cancer index
PFS	Progression-free survival
PMCA	Peritoneal mucinous carcinomatosis
PMCA-S	Peritoneal mucinous carcinomatosis with signet ring cells
PMP	Pseudomyxoma peritonei
PRISMA	Preferred reporting items for systematic reviews and meta-analyses
PSOGI	Peritoneal Surface Oncology Group International
REML	Restricted Maximum Likelihood
WHO	World Health Organization
