# Supplementary material for: The Role of Prognostic Clinical-Pathological, (Immuno-) Histological, and Molecular Parameters in Pseudomyxoma Peritonei Patients Undergoing Cytoreductive Surgery and Hyperthermic Intraperitoneal Chemotherapy: A Systematic Review and Meta-Analysis
Source: Cancers (Basel). 2026 Feb 28;18(5):795. doi: 10.3390/cancers18050795 (PMC12984246; doi:10.3390/cancers18050795)
Supplement: Supplementary file 1 [file cancers-18-00795-s001.zip › cancers-4129777-supplementary.pdf]

Table S1. Excluded studies due to missing hazard ratios for OS, PFS, or DFS.

No.	Author, Year	Study Design	Number of Patients	Primary Objective of the Study
1	Bangeas, P. et al., 2023 [1]	Retrospective, single-center	4	Clinicopathological features
2	Kepenekian, V. et al., 2022 [2]	Retrospective, multicenter	129	Clinical impact of scalloping of the liver and spleen
3	Bai, M. et al., 2022 [3]	Retrospective, single-center	144	Nomogram for prediction of incomplete cytoreduction
4	Lu, Y. et al., 2021 [4]	Retrospective, single-center	22	Clinicopathological features
5	Isella, C. et al., 2020 [5]	Retrospective, single-center	177	Transcriptional profiling
6	Tan, G. H. et al., 2017 [6]	Prospective, single-center	260	Application of the modified Glasgow prognosis
7	Di Fabio, F. et al., 2016 [7]	Retrospective, single-center	1014	The role of gastrectomy
8	Pietrantonio, F. et al., 2016 [8]	Prospective, single-center	15	The prognostic role of GNAS mutational status
9	Di Fabio, F. et al., 2015 [9]	Prospective, single-center	30	The prognostic role of elevated tumor markers
10	Lord, A. C. et al., 2015 [10]	Retrospective, single-center	512	Evaluation of the outcomes of re-do surgery
11	Pietrantonio, F. et al., 2014 [11]	Prospective, single-center	20	Assessment of the activity of chemotherapy with 5-fluorouracil and oxaliplatin (FOLFOX-4 regimen)
12	Taflampas, P. et al., 2014 [12]	Prospective, single-center	752	The prognostic role of elevated tumor markers
13	Wang, H. et al., 2014 [13]	Retrospective, single-center	39	Clinicopathological features
14	Low, R. N. et al., 2013 [14]	Retrospective, single-center	50	Surveillance strategy

Table S2. The quality of the included studies (Newcastle-Ottawa Quality Assessment Scale).

		Selection				Comparability	Outcome			NOS Score
**No.**	**Author, Year**	**Representativeness of the exposed cohort**	**Selection of the non-exposed cohort**	**Ascertainment of exposure**	**Outcome of interest not present at start of study**	**Comparability of cohorts based on the design or analysis**	**Assessment of outcome**	**Sufficient follow-up**	**Adequacy of follow- up**	
1	Chandrakumaran, K. et al. [15]	*		*	*	*	*	*	*	7
2	Blaj, S. et al. [16]	*		*	*		*	*		5
3	Nizam, W. et al. [17]	*		*	*		*	*	*	6
4	Ma, R. et al. [18]			*	*		*		*	4
5	Solomon, D. et al. [19]	*		*	*		*	*	*	6

6	Van Eden, W.J. et al. 2019 [20]	*		*	*		*	*	*	6
7	Rangarajan, K. et al. 2018 [21]	*		*	*		*	*	*	6
8	Pietrantonio, F. et al. 2016 [22]			*	*		*	*	*	5
9	Kusamura, S. et al. 2016 [23]	*		*	*		*	*	*	6
10	Kusamura, S. et al. 2015 [24]	*		*	*		*		*	5
11	Kusamura, S. et al. 2013 [25]	*		*	*		*	*	*	6
12	Canbay, E. et al. 2013 [26]	*		*	*		*	*	*	6
13	Baratti, D. et al. 2009 [27]	*		*	*		*	*	*	6
14	Elias, D. et al. 2008 [28]	*		*	*		*	*	*	6
15	Baratti, D. et al. 2007 [29]			*	*		*	*	*	5
16	Van Ruth, S. et al. 2002 [30]			*	*		*		*	4
Summary										6[1]<br>5,6[2]

[1]: Median, [2]: Mean.

Table S3. Grading of Recommendations, Assessment, Development and Evaluation (GRADE) Working Group grades of evidence of the included studies.

No.	Author, Year	Quality of the evidence (GRADE)
1	Chandrakumaran, K. et al. [15]	High
2	Blaj, S. et al. [16]	Moderate
3	Nizam, W. et al. [17]	Moderate
4	Ma, R. et al. [18]	Low
5	Solomon, D. et al. [19]	Moderate
6	Van Eden, W.J. et al. 2019 [20]	Moderate
7	Rangarajan, K. et al. 2018 [21]	Moderate
8	Pietrantonio, F. et al. 2016 [22]	Moderate
9	Kusamura, S. et al. 2016 [23]	Moderate
10	Kusamura, S. et al. 2015 [24]	Moderate
11	Kusamura, S. et al. 2013 [25]	Moderate
12	Canbay, E. et al. 2013 [26]	Moderate
13	Baratti, D. et al. 2009 [27]	Moderate
14	Elias, D. et al. 2008 [28]	Moderate
15	Baratti, D. et al. 2007 [29]	Moderate
16	Van Ruth, S. et al. 2002 [30]	Low

Table S4. Trim-and-fill sensitivity analysis results.

Analysis	Original k	Original HR [95% CI]	Trim-Fill k	Trim-Fill HR [95% CI]	Imputed Studies	Egger's t (p-value)
**OS univariate**	28	2.29 [1.76-2.97]	36	1.78 [1.36-2.32]	8 left	12.22 ($p < 0.0001$)
**OS multivariate**	36	2.32 [1.86-2.89]	50	1.66 [1.29-2.13]	14 left	13.76 ($p < 0.0001$)
**PFS univariate**	15	1.80 [1.29-2.51]	21	1.32 [0.93-1.88]	6 left	12.07 ($p < 0.0001$)
**PFS multivariate**	15	2.32 [1.67-3.22]	21	1.75 [1.27-2.41]	6 left	10.76 ($p < 0.0001$)
**DFS univariate**	29	2.42 [1.73-3.38]	No studies imputed		0	8.59 ($p < 0.0001$)
**DFS multivariate**	15	2.35 [1.46-3.78]	No studies imputed		0	7.42 ($p < 0.0001$)

HR, hazard ratio; CI, confidence interval.

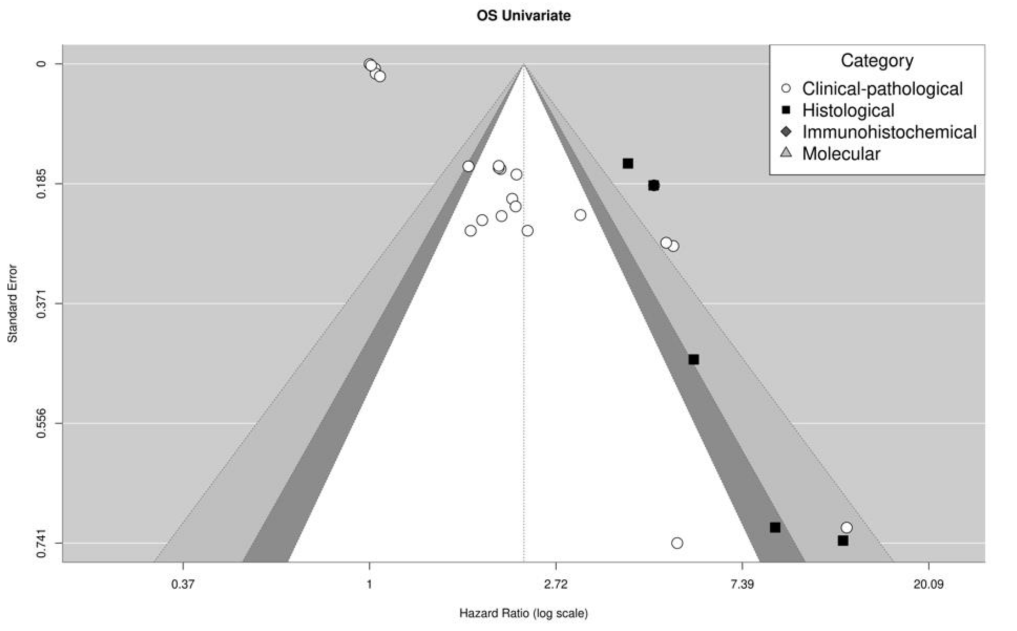

(a)

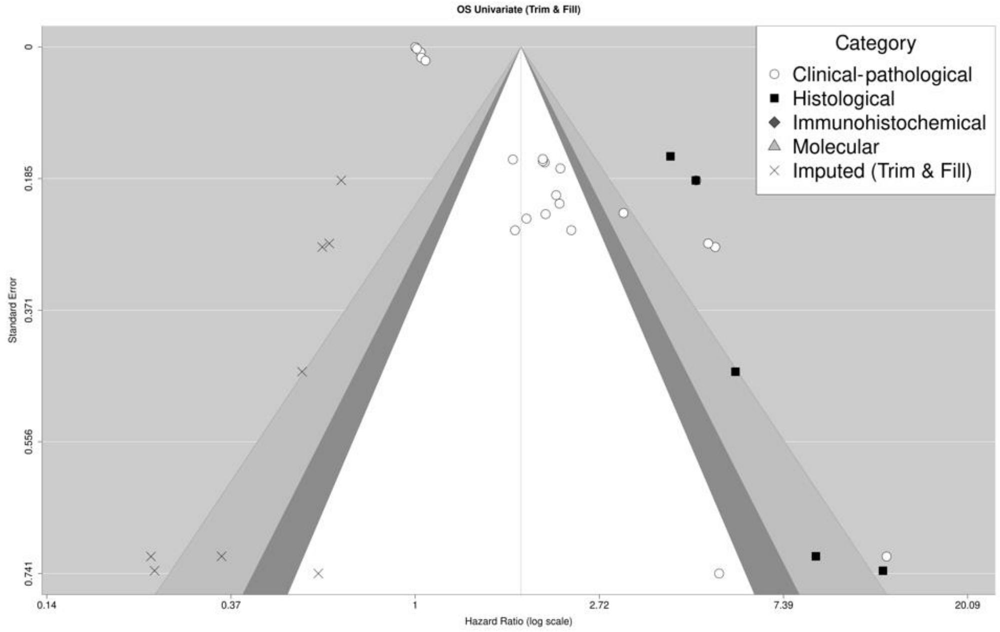
OS Univariate (Trim & Fill)
Category
Clinical-pathological
Histological
Immunohistochemical
Molecular
Imputed (Trim & Fill)
Standard Error
0
0.185
0.371
0.556
0.741
0.14
0.37
1
2.72
7.39
20.09
Hazard Ratio (log scale)

(b)

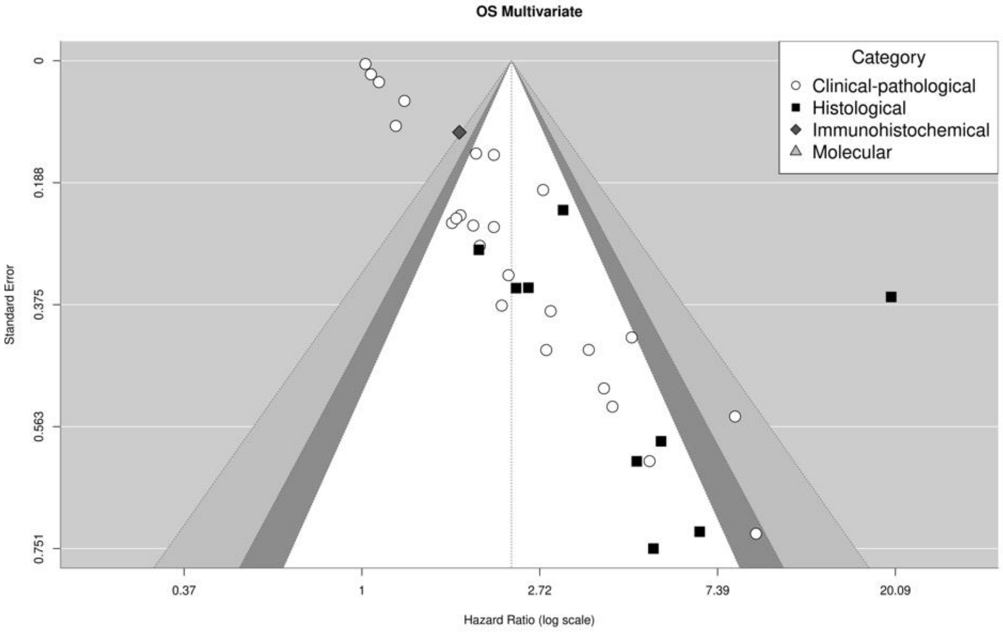
OS Multivariate
Category
Clinical-pathological
Histological
Immunohistochemical
Molecular
Standard Error
0
0.188
0.375
0.563
0.751
0.37
1
2.72
7.39
20.09
Hazard Ratio (log scale)

(c)

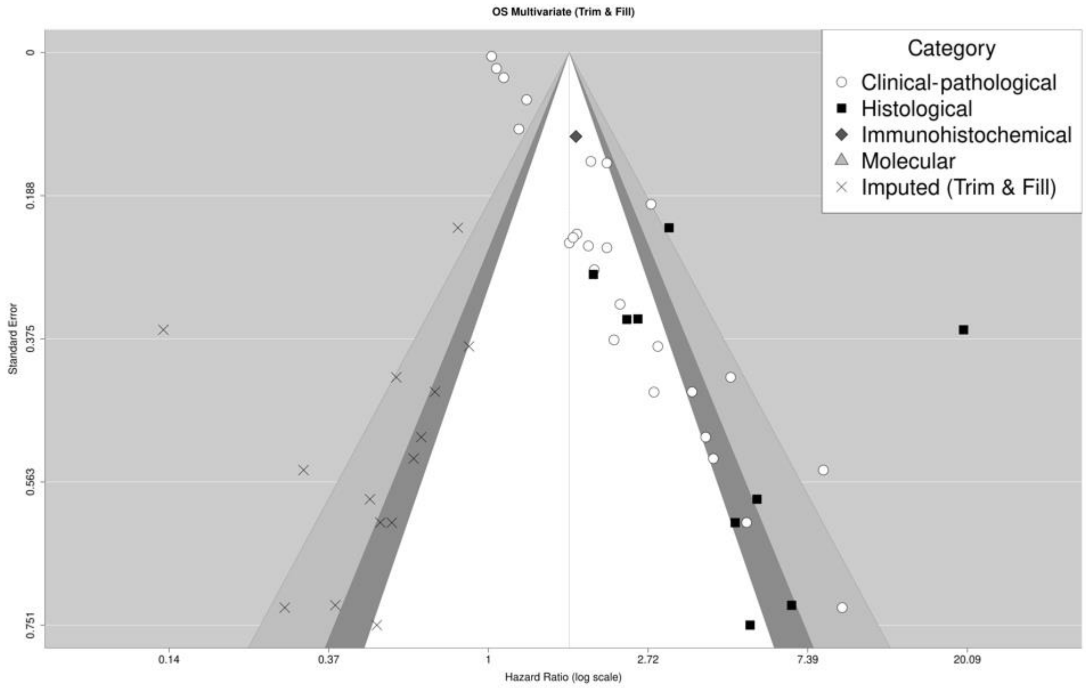

(d)

Figure S1. Funnel plot of studies examining the association between clinical-pathological, histological, and immunohistochemical parameters with overall survival in univariate (a), trim-and-fill adapted univariate (b), multivariate (c), and trim-and-fill adapted multi-variate (d) analysis.

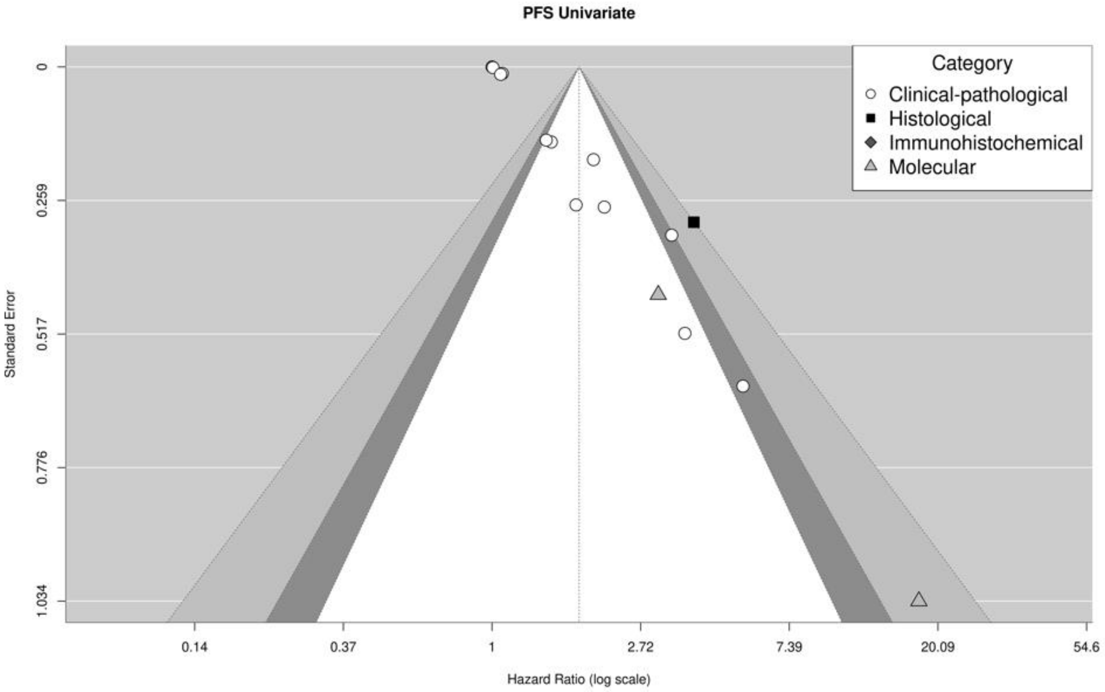

(a)

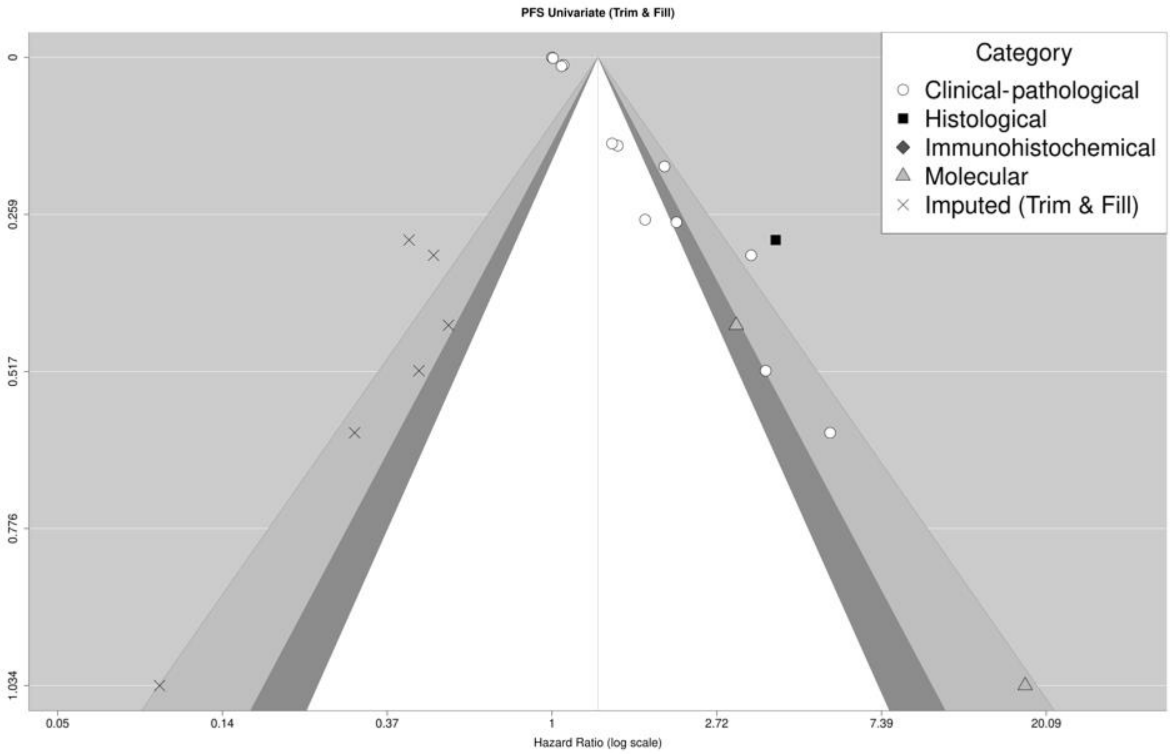
PFS Univariate (Trim & Fill)
Category
Clinical-pathological
Histological
Immunohistochemical
Molecular
Imputed (Trim & Fill)
0
0.259
0.517
0.776
1.034
0.05
0.14
0.37
1
2.72
7.39
20.09
Hazard Ratio (log scale)

(b)

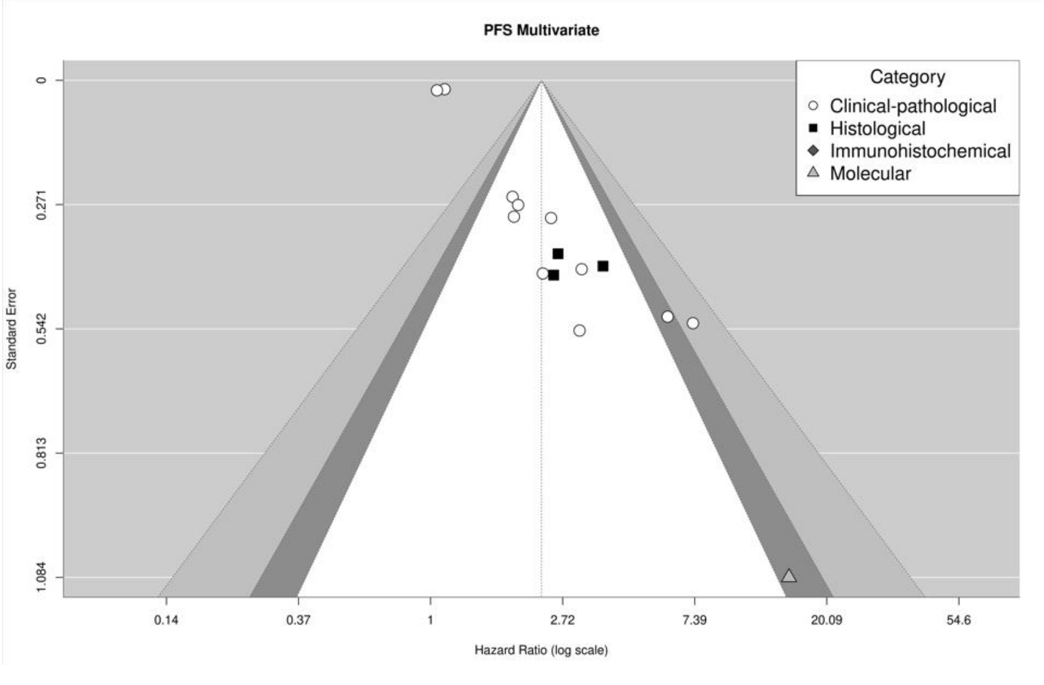
PFS Multivariate
Category
Clinical-pathological
Histological
Immunohistochemical
Molecular
Standard Error
0
0.271
0.542
0.813
1.084
0.14
0.37
1
2.72
7.39
20.09
54.6
Hazard Ratio (log scale)

(c)

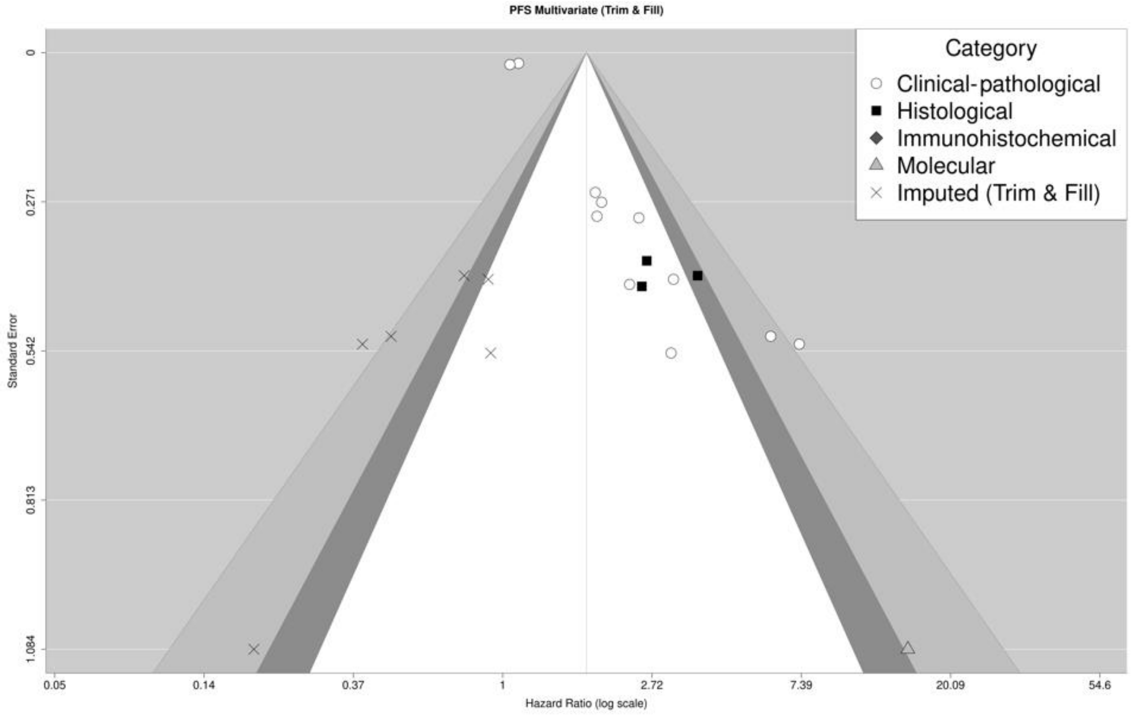

(d)

Figure S2. Funnel plot of studies examining the association between clinical-pathological, histological, and molecular parameters with progression-free survival in univariate (a), trim-and-fill adapted univariate (b), multivariate (c), and trim-and-fill adapted multivariate (d) analysis;.

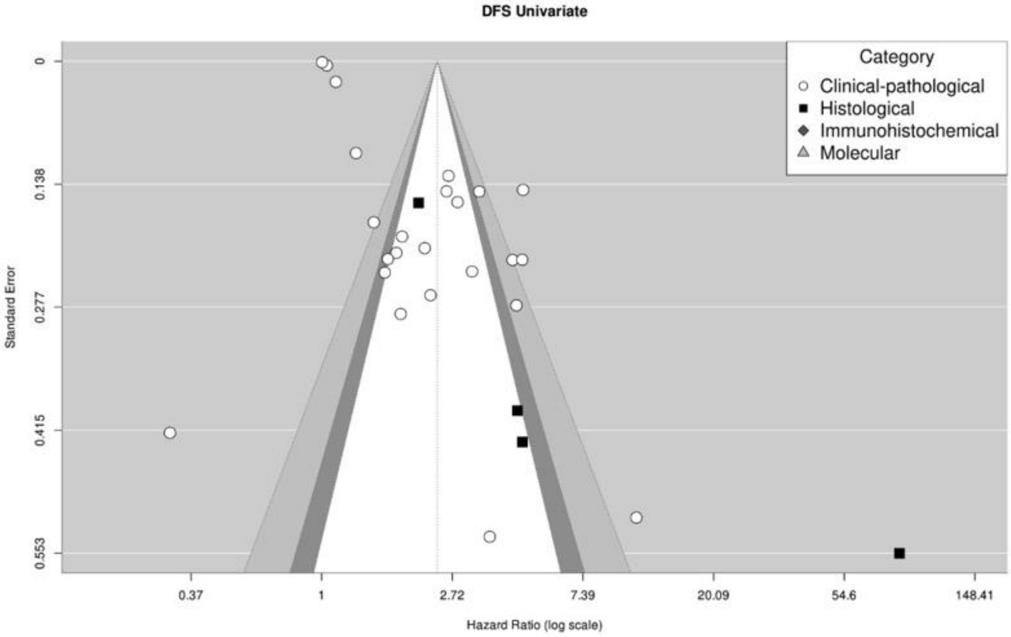

(a)

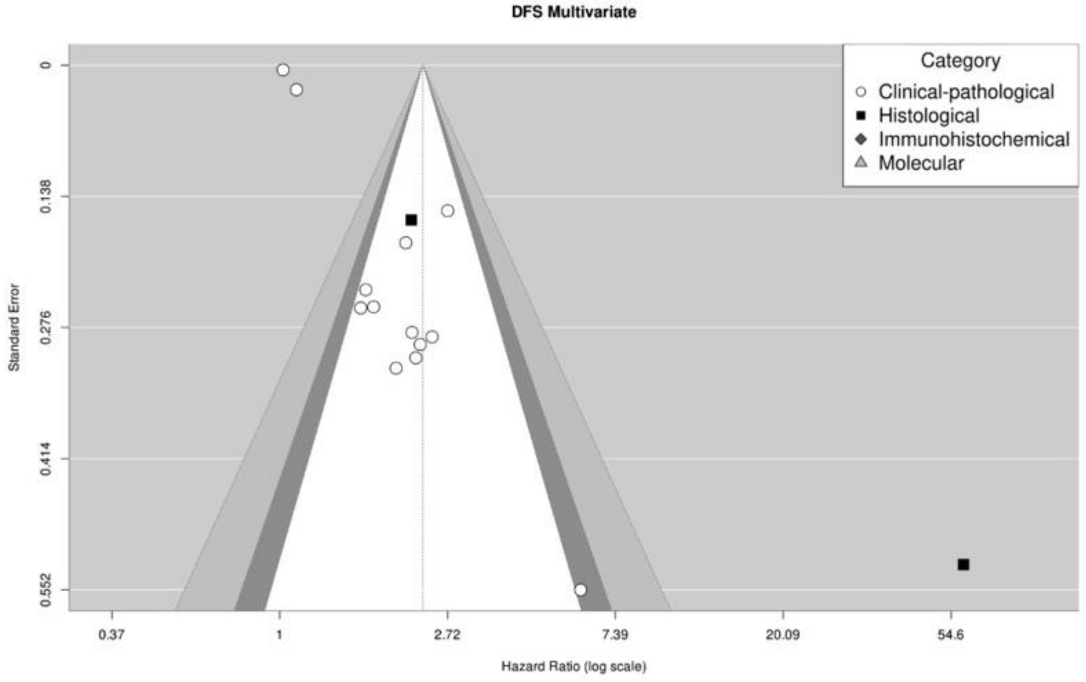

(b)

Figure S3. Funnel plot of studies examining the association between clinical-pathological and histological parameters with disease-free survival in univariate (a) and multivariate (b) analysis.

References

1. Bangeas P, Kyziridis D, Kalakonas A, et al. Low-Grade Pseudomyxoma Peritonei Behaving as a High-Grade Disease: A Case Series and Literature Review. Curr Oncol. 2023;30(11):9996-10006. doi: 10.3390/curroncol30110726
2. Kepenekian V, Kefleyesus A, Keskin D, et al. Scalloping of the Liver and Spleen on Preoperative CT-Scan of Pseudomyxoma Peritonei Patients: Impact on Prediction of Resectability, Grade, Morbidity and Survival. Cancers (Basel). 2022;14(18). doi: 10.3390/cancers14184434
3. Bai M, Wang S, Liang G, et al. Nomogram to Predict Incomplete Cytoreduction for Pseudomyxoma Peritonei. Ann Surg Oncol. 2022;29(2):885-892. doi: 10.1245/s10434-021-10725-4
4. Lu Y, Li F, Ma R, et al. Clinicopathological Features of Low-Grade Appendiceal Mucinous Neoplasms Confined to the Appendix. Front Oncol. 2021;11696846. doi: 10.3389/fonc.2021.696846
5. Isella C, Vaira M, Robella M, et al. Improved Outcome Prediction for Appendiceal Pseudomyxoma Peritonei by Integration of Cancer Cell and Stromal Transcriptional Profiles. Cancers (Basel). 2020;12(6). doi: 10.3390/cancers12061495
6. Tan GH, Novo CA, Dayal S, et al. The modified Glasgow prognosis score predicts for overall and disease-free survival following cytoreductive surgery and HIPEC in patients with pseudomyxoma peritonei of appendiceal origin. Eur J Surg Oncol. 2017;43(2):388-394. doi: 10.1016/j.ejso.2016.10.009
7. Di Fabio F, Mehta A, Chandrakumaran K, et al. Advanced Pseudomyxoma Peritonei Requiring Gastrectomy to Achieve Complete Cytoreduction Results in Good Long-Term Oncologic Outcomes. Ann Surg Oncol. 2016;23(13):4316-4321. doi: 10.1245/s10434-016-5389-7
8. Pietrantonio F, Berenato R, Maggi C, et al. GNAS mutations as prognostic biomarker in patients with relapsed peritoneal pseudomyxoma receiving metronomic capecitabine and bevacizumab: a clinical and translational study. J Transl Med. 2016;14(1):125. doi: 10.1186/s12967-016-0877-x

9. Di Fabio F, Aston W, Mohamed F, et al. Elevated tumour markers are normalized in most patients with pseudomyxoma peritonei 7 days after complete tumour removal. Colorectal Dis. 2015;17(8):698-703. doi: 10.1111/codi.12924
10. Lord AC, Shihab O, Chandrakumaran K, et al. Recurrence and outcome after complete tumour removal and hyperthermic intraperitoneal chemotherapy in 512 patients with pseudomyxoma peritonei from perforated appendiceal mucinous tumours. Eur J Surg Oncol. 2015;41(3):396-399. doi: 10.1016/j.ejso.2014.08.476
11. Pietrantonio F, Maggi C, Fanetti G, et al. FOLFOX-4 chemotherapy for patients with unresectable or relapsed peritoneal pseudomyxoma. Oncologist. 2014;19(8):845-850. doi: 10.1634/theoncologist.2014-0106
12. Taflampas P, Dayal S, Chandrakumaran K, et al. Pre-operative tumour marker status predicts recurrence and survival after complete cytoreduction and hyperthermic intraperitoneal chemotherapy for appendiceal Pseudomyxoma Peritonei: Analysis of 519 patients. Eur J Surg Oncol. 2014;40(5):515-520. doi: 10.1016/j.ejso.2013.12.021
13. Wang H, Wang X, Ju Y, et al. Clinicopathological features and prognosis of pseudomyxoma peritonei. Exp Ther Med. 2014;7(1):185-190. doi: 10.3892/etm.2013.1408
14. Low RN, Barone RM and Lee MJ. Surveillance MR imaging is superior to serum tumor markers for detecting early tumor recurrence in patients with appendiceal cancer treated with surgical cytoreduction and HIPEC. Ann Surg Oncol. 2013;20(4):1074-1081. doi: 10.1245/s10434-012-2788-2
15. Chandrakumaran, K.; Carr, N.J.; Mohamed, F.; Cecil, T.D.; Moran, B.J. Development and Validation of Nomograms to Predict Survival in Patients Undergoing Complete Cytoreduction and Hyperthermic Intraperitoneal Chemotherapy for Pseudomyxoma Peritonei of Appendiceal Origin. JAMA Surg. 2023, 158, 522–530. https://doi.org/10.1001/jamasurg.2023.0112.
16. Blaj, S.; Dora, D.; Lohinai, Z.; Herold, Z.; Szasz, A.M.; Herzberg, J.; Kodacsi, R.; Baransi, S.; Schlitt, H.J.; Hornung, M.; et al. Prognostic Factors in Pseudomyxoma Peritonei with Emphasis on the Predictive Role of Peritoneal Cancer Index and Tumor Markers. Cancers 2023, 15, 1326. https://doi.org/10.3390/cancers15041326.
17. Nizam, W.; Fackche, N.; Pessoa, B.; Kubi, B.; Cloyd, J.M.; Grotz, T.; Fournier, K.; Dineen, S.; Veerapong, J.; Baumgartner, J.M.; et al. Prognostic Significance of Preoperative Tumor Markers in Pseudomyxoma Peritonei from Low-Grade Appendiceal Mucinous Neoplasm: A Study from the US HIPEC Collaborative. J. Gastrointest. Surg. 2022, 26, 414–424. https://doi.org/10.1007/s11605-021-05075-1.
18. Ma, R.; Lin, Y.L.; Li, X.B.; Yan, F.C.; Xu, H.B.; Peng, Z.; Li, Y. Tumor-stroma ratio as a new prognosticator for pseudomyxoma peritonei: A comprehensive clinicopathological and immunohistochemical study. Diagn. Pathol. 2021, 16, 116. https://doi.org/10.1186/s13000-021-01177-1.
19. Solomon, D.; Bekhor, E.; Leigh, N.; Maniar, Y.M.; Totin, L.; Hofstedt, M.; Aycart, S.N.; Carr, J.; Ballentine, S.; Magge, D.R.; et al. Surveillance of Low-Grade Appendiceal Mucinous Neoplasms with Peritoneal Metastases After Cytoreductive Surgery and Hyperthermic Intraperitoneal Chemotherapy: Are 5 Years Enough? A Multisite Experience. Ann. Surg. Oncol. 2020, 27, 147–153. https://doi.org/10.1245/s10434-019-07678-0.
20. van Eden, W.J.; Kok, N.F.M.; Snaebjornsson, P.; Jóźwiak, K.; Woensdregt, K.; Bottenberg, P.D.; Boot, H.; Aalbers, A.G.J. Factors influencing long-term survival after cytoreductive surgery and hyperthermic intraperitoneal chemotherapy for pseudomyxoma peritonei originating from appendiceal neoplasms. BJS Open 2019, 3, 376–386. https://doi.org/10.1002/bjs5.50134.
21. Rangarajan, K.; Chandrakumaran, K.; Dayal, S.; Mohamed, F.; Moran, B.J.; Cecil, T.D. The pre-operative neutrophil-lymphocyte ratio predicts overall and disease-free survival following cytoreductive surgery (CRS) and hyperthermic intraperitoneal chemotherapy (HIPEC) in patients with pseudomxyoma peritonei of appendiceal origin. Int. J. Hyperth. 2018, 34, 559–563. https://doi.org/10.1080/02656736.2017.1384073.
22. Pietrantonio, F.; Perrone, F.; Mennitto, A.; Gleeson, E.M.; Milione, M.; Tamborini, E.; Busico, A.; Settanni, G.; Berenato, R.; Caporale, M.; et al. Toward the molecular dissection of peritoneal pseudomyxoma. Ann. Oncol. 2016, 27, 2097–2103. https://doi.org/10.1093/annonc/mdw314.
23. Kusamura, S.; Torres Mesa, P.A.; Cabras, A.; Baratti, D.; Deraco, M. The Role of Ki-67 and Pre-cytoreduction Parameters in Selecting Diffuse Malignant Peritoneal Mesothelioma (DMPM) Patients for Cytoreductive Surgery (CRS) and Hyperthermic Intraperitoneal Chemotherapy (HIPEC). Ann. Surg. Oncol. 2016, 23, 1468–1473. https://doi.org/10.1245/s10434-015-4962-9.
24. Kusamura, S.; Baratti, D.; Hutanu, I.; Gavazzi, C.; Morelli, D.; Iusco, D.R.; Grassi, A.; Bonomi, S.; Virzì, S.; Haeusler, E.; et al. The role of baseline inflammatory-based scores and serum tumor markers to risk stratify pseudomyxoma peritonei patients treated with cytoreduction (CRS) and hyperthermic intraperitoneal chemotherapy (HIPEC). Eur. J. Surg. Oncol. 2015, 41, 1097–1105. https://doi.org/10.1016/j.ejso.2015.04.005.
25. Kusamura, S.; Hutanu, I.; Baratti, D.; Deraco, M. Circulating tumor markers: Predictors of incomplete cytoreduction and powerful determinants of outcome in pseudomyxoma peritonei. J. Surg. Oncol. 2013, 108, 1–8. https://doi.org/10.1002/jso.23329.

26. Canbay, E.; Ishibashi, H.; Sako, S.; Mizumoto, A.; Hirano, M.; Ichinose, M.; Takao, N.; Yonemura, Y. Preoperative carcinoembryonic antigen level predicts prognosis in patients with pseudomyxoma peritonei treated with cytoreductive surgery and hyperthermic intraperitoneal chemotherapy. World J. Surg. 2013, 37, 1271–1276. https://doi.org/10.1007/s00268-013-1988-7.
27. Baratti, D.; Kusamura, S.; Nonaka, D.; Cabras, A.D.; Laterza, B.; Deraco, M. Pseudomyxoma peritonei: Biological features are the dominant prognostic determinants after complete cytoreduction and hyperthermic intraperitoneal chemotherapy. Ann. Surg. 2009, 249, 243–249. https://doi.org/10.1097/SLA.0b013e31818eec64.
28. Elias, D.; Honoré, C.; Ciuchendéa, R.; Billard, V.; Raynard, B.; Lo Dico, R.; Dromain, C.; Duvillard, P.; Goéré, D. Peritoneal pseudomyxoma: Results of a systematic policy of complete cytoreductive surgery and hyperthermic intraperitoneal chemotherapy. Br. J. Surg. 2008, 95, 1164–1171. https://doi.org/10.1002/bjs.6235.
29. Baratti, D.; Kusamura, S.; Martinetti, A.; Seregni, E.; Laterza, B.; Oliva, D.G.; Deraco, M. Prognostic value of circulating tumor markers in patients with pseudomyxoma peritonei treated with cytoreductive surgery and hyperthermic intraperitoneal chemotherapy. Ann. Surg. Oncol. 2007, 14, 2300–2308. https://doi.org/10.1245/s10434-007-9393-9.
30. van Ruth, S.; Hart, A.A.; Bonfrer, J.M.; Verwaal, V.J.; Zoetmulder, F.A. Prognostic value of baseline and serial carcinoembryonic antigen and carbohydrate antigen 19.9 measurements in patients with pseudomyxoma peritonei treated with cytoreduction and hyperthermic intraperitoneal chemotherapy. Ann. Surg. Oncol. 2002, 9, 961–967. https://doi.org/10.1007/bf02574513.
